# Editorial: Biotechnological applications of endophytes in agriculture, environment and industry

**DOI:** 10.3389/fmicb.2023.1269279

**Published:** 2023-08-15

**Authors:** Haiyan Li, Vijay K. Sharma, George Newcombe, Daniela Barretto Barbosa Trivella, Ravindra Soni

**Affiliations:** ^1^Medical School, Kunming University of Science and Technology, Kunming, China; ^2^Agricultural Research Organization, The Volcani Center, Rishon LeZion, Israel; ^3^Department of Forest, Rangeland and Fire Sciences, University of Idaho, Moscow, ID, United States; ^4^Brazilian Biosciences National Laboratory, National Center for Research in Energy and Materials, Campinas, SP, Brazil; ^5^Department of Agricultural Microbiology, College of Agriculture, Indira Gandhi Krishi Vishwavidyalya, Raipur, Chhattisgarh, India

**Keywords:** endophytes, agriculture, environment, industry, biotechnological applications

Endophytes are the plant symbionts that live inside the plant tissue without causing any symptoms of disease for a part of their life-cycle. They are an important untapped reservoir of biological resources. They can promote plant growth by improving the physiological and metabolic functions of host plants via nutrient acquisition, nitrogen fixation, phytohormone production, etc., which can be used to promote agricultural yield and food quality. They also have potential applications in enhanced phytoremediation. In addition, endophytes are known to produce various novel antibiotics that can be used in the pharmaceutical, food, and agricultural industries. Functional genomics studies of endophytes provided more information and a better understanding of the network of complex host-endophyte interactions and other associated microbes to harness the biotechnological potential of endophytes more efficiently and sustainably.

The main aim of this Research Topic was to recover the functional role and application of endophytes for agricultural, medicinal, industrial, and environmental purposes. Within this topic, nine articles have been published that complement our knowledge on the occurrence and diversity of endophytes and the role, mechanism, and biotechnological application of endophytes in these fields.

One of the main causes of the global drop in crop productivity is pathogenic microorganisms. Endophytes diminish the injury triggered by pathogens through synthesizing antibiosis, the production of lytic enzymes, secondary metabolites, hormone activation, etc. (Chaudhary et al.). An et al. isolated an endophytic bacterium *Burkholderia ambifaria* XN08 with antagonistic activity against *Rhizoctonia cerealis*, a wheat (*Triticum aestivum* L.) sharp eyespot pathogenic fungus. The colonization of strain XN08 was accompanied by an enhancement of wheat growth and an induction of wheat sharp eyespot resistance by synthesizing a series of plant growth regulators (indole-3-acetic acid, IAA, etc.), producing antifungal compounds (pyrrolnitrin, etc.), and enhancing the activities of defense enzymes (polyphenol oxidase, peroxidase, and phenylalanine ammonia-lyase). The role of *Trichoderma asperellum* against *Fusarium* wilt disease (FDW) in tomato (*Solanum lycopersicum*) plants was investigated by Sehim et al.. They found that *T. asperellum* exhibited the highest mycelial inhibition rate (53.24%) against *Fusarium oxysporum*. *T. asperellum* enhanced the growth of tomato seeds and controlled the FDW by enhancing the number of leaves, as well as shoot and root length and fresh and dry weights by producing IAA, Phosphate (P) solubilization, and synthesizing bioactive secondary metabolites. Furthermore, *Trichoderma* extract increased shelf-life of tomato fruits by reducing infection by *F. oxysporum* from post-harvest.

Abiotic stress, such as drought and flood stress, heavy metal stress, prevents plants from growing normally and lowers crop output. Endophytes represent safe and effective biological agents that mitigate abiotic stress for plant development. Ou et al. screened out *Klebsiella aerogenes* HGG15 from 28 endophytic bacteria as having superior plant growth promotion (PGP) traits, including P solubilization, IAA, siderophore, and acetoin production, as well as biosafety for silkworms. Flood tolerance of mulberry (*Morus alba* L.) was increased by inoculated *K. aerogenes* HGG15 by synthesizing a series of abiotic stress response factors and growth promotion stimulators such as glycerolipid, sphingolipid, indole, pyridine, and coumarin. Santra and Banerjee isolated a Galactose-Rich Heteropolysaccharide (GRH) from endophytic *Mucor* sp. HELF2. Spraying with 50 ppm GRH has alleviated drought stress in rice seedlings (*Oryza sativa* ssp. indica MTU 7093 swarna) by improving relative water content and fresh weight of the tissues, root length, and shoot length, as well as increasing the soluble sugars, prolines, and chlorophyll contents of rice seedlings and elevating the enzymatic antioxidant parameters. The role of seed endophyte FXZ2 on *Dysphania ambrosioides* Zn/Cd tolerance and accumulation was investigated by Sharma et al.. The study suggests that the Zn/Cd tolerance of the host plant was increased by seed endophyte FXZ2 by altering Zn/Cd speciation in rhizospheric soils and exogenous production of phytohormones to promote growth, lowering oxidative damage while enhancing antioxidant properties. In addition, Zn uptake in inoculated plants was decreased, while Cd accumulation was increased in the inoculated plants that were grown in Zn/Cd contaminated soil. Similarly, Flores-Duarte et al. isolated and selected 4 endophytic rhizobia and non-rhizobia with higher PGP properties and bacterial enzymatic activities from *Medicago* spp., including *Pseudomonas* sp. N4, *Pseudomonas* sp. N8, *Ensifer* sp. N10 and *Ensifer* sp. N12. Inoculation with combinations of *Ensifer* (rhizobia) and *Pseudomonas* increased plant biomass and nodules ameliorating the physiological state of the plants and helping to regulate plant stress mechanisms, while increasing As, Cd, Cu, and Zn accumulation in plant roots, without significant differences in shoot metal accumulation, on nutrient-poor soils and moderately contaminated with metals/loids. Endophytes provide new insights into agricultural production and environmental health.

In the field of livestock feed production such as silage, microbes with antibacterial and other properties have been extensively researched and used. Zhang et al. assessed the effects of antibacterial peptide-producing *Bacillus subtilis* CP7 on the fermentation quality and bacterial community of different varieties of whole-plant corn silage. The additive *B. subtilis* CP7 enhanced the quantity of dry matter and crude protein, and improved the structure of the bacterial community following silage.

With the development of technology, artificial intelligence (AI) has been extensively used in the biotechnology and applied microbiology sectors. Deep learning, prediction, support vector machines, object detection, feature representation, synthetic biology, amyloid, human microRNA precursors, systems biology, and single cell RNA-Sequencing were the current hot spots, while microRNA and protein-protein interactions (PPIs) are the future trends in this area (Xu et al.). Studying PPIs using AI methods provides a better understanding of the complex network of host-endophyte interactions and other associated microbes to harness the biotechnological potential of endophytes more efficiently and sustainably.

In conclusion, endophytes were developed as an eco-friendly microbial agent for overcoming the tasks faced with conventional farming, the environment, and industry. Coupled with the AI, microbiome, and metabolite analyses, the mechanism of the role of endophytes could possibly be studied effectively and deeply, consequently amplifying the application potential of these beneficial microbes.

## Author contributions

HL: Writing—original draft, Writing—review and editing. VS: Writing—review and editing. GN: Writing—review and editing. DT: Writing—review and editing. RS: Writing—review and editing.

